# ATP Hydrolyzing Salivary Enzymes of Caterpillars Suppress Plant Defenses

**DOI:** 10.1371/journal.pone.0041947

**Published:** 2012-07-25

**Authors:** Shuang Wu, Michelle Peiffer, Dawn S. Luthe, Gary W. Felton

**Affiliations:** 1 Department of Entomology and Center for Chemical Ecology, Pennsylvania State University, University Park, Pennsylvania, United States of America; 2 Department of Crop and Soil Science and Center for Chemical Ecology, Pennsylvania State University, University Park, Pennsylvania, United States of America; 3 Key Laboratory of Entomology and Pest Control Engineering, College of Plant Protection, Southwest University, Chongqing, P. R. China; United States Department of Agriculture, Agriculture Research Service, United States of America

## Abstract

The oral secretions of herbivores are important recognition cues that can be used by plants to mediate induced defenses. In this study, a degradation of adenosine-5′-triphosphate (ATP) in tomato leaves was detected after treatment with *Helicoverpa zea* saliva. Correspondingly, a high level of ATPase activity in saliva was detected and three ATP hydrolyzing enzymes: apyrase, ATP synthase and ATPase 13A1 were identified in salivary glands. To determine the functions of these proteins in mediating defenses, they were cloned from *H. zea* and expressed in *Escherichia coli*. By applying the purified expressed apyrase, ATP synthase or ATPase 13A1 to wounded tomato leaves, it was determined that these ATP hydrolyzing enzymes suppressed the defensive genes regulated by the jasmonic acid and ethylene pathways in tomato plant. Suppression of glandular trichome production was also observed after treatment. Blood-feeding arthropods employ 5′-nucleotidase family of apyrases to circumvent host responses and the *H. zea* apyrase, is also a member of this family. The comparatively high degree of sequence similarity of the *H. zea* salivary apyrase with mosquito apyrases suggests a broader evolutionary role for salivary apyrases than previously envisioned.

## Introduction

The oral secretions of herbivores have been reported to be important cues in mediating inducible plant responses [1]–[12]. Plants generate inducible defense responses according to the overall effect of elicitors and effectors from caterpillar oral secretions. In some cases, an effector/elicitor from saliva could cause different responses in different host plants. Our previous studies showed that saliva suppresses induced resistance in tobacco *Nicotiana tabacum* [7], [11], and glucose oxidase (GOX), an abundant protein in the saliva of the caterpillar *Helicoverpa zea* that suppresses nicotine induction was identified [7]. Glucose oxidase has since been shown to be present in many caterpillars [12]–[15] and in the caterpillar *Spodoptera exigua* where it suppresses the jasmonic acid pathway in *Nicotiana attenuata* [12]. Recently, it was reported that *H. zea* salivary glucose oxidase has opposing effects on tomato *Solanum lycopersicum*. It elicits both rapid and delayed-induced defenses in tomato plants [10]. Nevertheless, saliva contains scores of proteins many of which have no known function [10].

Most parasites deploy a cocktail of effectors with multiple and overlapping functions to suppress host responses [16], [17]. One of the nearly universal mechanisms employed to suppress host responses by blood-feeding arthropods such as mosquitoes and ticks is to suppress platelet aggregation by the enzymatic hydrolysis of extracellular adenosine diphosphate by apyrases [18]–[20]. Apyrase is nucleoside triphosphate-diphosphohydrolase (EC 3.6.1.5) that catalyzes the hydrolysis of ATP and ADP to yield AMP and inorganic phosphate. It has not been reported if insect herbivores possess salivary apyrase. This is relevant because in plants, ATP can be released from cells by plasma membrane ABC transporters [21], [22], vesicular efflux [23] and during wounding [24]. During the last decade, extracellular ATP (eATP) has been shown to be a vital factor in regulating a wide range of responses such as cell viability and death [25], growth, development [22], [23], [26]–[29], and stress responses [21], [24]. Jeter et al. [24] detected that ATP-induced increases in cytoplasmic calcium levels were associated with downstream gene expression. Chivasa et al. [25] showed that defenses in *Arabidopsis* were activated by fumonisin B1, a programmed cell death-eliciting mycotoxin, or other enzymes that degraded ATP (e.g. apyrase). In tobacco, Chivasa et al. [30] showed that enzymatic depletion of eATP by apyrase induced pathogenesis-related (PR) gene expression and enhanced resistance to tobacco mosaic virus and *Pseudomonas syringae pv. tabaci*. Thus, eATP serves as a *negative* regulator of systemic acquired resistance. In plants, apyrases play an important role in the regulation of eATP [21], [28], [29].

Following our observation that foliar levels of ATP in tomato declined after exposure to caterpillar saliva, several salivary enzymes that hydrolyze ATP were identified in this study, and the genes were cloned and expressed in *E. coli*. Then, their effects on defenses in tomato were determined.

## Results

### Saliva depletes foliar levels of ATP

Based on the importance of eATP in plant physiological responses, *H. zea* saliva was assayed for ATP hydrolysis activity. By adding 5 µl diluted (30×) plant leaf apoplastic fluid, which contained 0.1 µM eATP, to cuvette and reacted with 5 µl saliva for 5 min and using the 100 µl rL/L Reagent, 93.9±3.4 nmol·min^−1^·mg^−1^ ATP hydrolysis specific activity was detected in 0.2±0.0 µg·µl^−1^ saliva (Table 1), and it degraded 0.4±0.0 nM eATP from tomato leaves.

**Table 1:** Comparison of the ATP hydrolysis activities of purified apyrase, ATP synthase and ATPase 13A1 from *E. coli*.*

		ATP hydrolysis activity			
Protein	Protein concentration (µg·µl^−1^)	Total activity (pmol·L^−1^·min^−1^)	Specific activity (nmol·min^−1^·mg^−1^)	Purification (-fold)	Yield (%)
saliva	0.2±0.0	74.3±0.0	93.9±3.4	-	-
apyrase^1^	1.0±0.0 a	707.3±3.5 a	139.4±1.8 a	1.7±0.0 a	73.8±0.4 a
apyrase^2^	1.1±0.0 ab	448.3±12.0 b	83.7±1.8 b	1.1±0.0 b	48.1±1.3 b
apyrase^3^	1.1±0.0 b	1062.9±4.5 c	196.5±5.0 c	2.5±0.1 c	111.9±0.5 c
ATP synthase^1^	1.3±0.0 a	774.3±3.5 a	116.9±1.5 a	0.8±0.0 a	100.0±0.5 a
ATP synthase^2^	1.6±0.0 b	743.5±5.4 a	93.3±1.8 b	0.6±0.0 b	96.5±0.7 a
ATP synthase^3^	1.6±0.0 b	1798.8±32.8 b	219.8±6.4 c	1.6±0.1 c	231.5±4.2 b
ATPase 13A1^1^	1.1±0.0 a	244.0±6.1 a	46.4±0.9 a	0.3±0.0 a	29.6±0.7 a
ATPase 13A1^2^	1.3±0.0 b	380.1±2.5 b	57.6±1.4 b	0.3±0.0 b	43.9±0.3 b
ATPase 13A1^3^	1.4±0.0 c	583.9±11.2 c	83.7±1.6 c	0.5±0.0 c	70.4±1.4 c
crude apyrase	2.4±0.0	946.9±7.8	79.7±0.5	-	-
crude ATP synthase	1.1±0.0	774.0±1.9	144.3±3.0	-	-
crude ATPase 13A1	1.0±0.0	840.0±12.9	162.5±3.2	-	-
crude vector protein	4.7±0.1	80.1±3.5	3.4±0.2	-	-

* The numbers following each name of the proteins represent that the proteins were obtained from different steps of the secondary purification. Each value represents the mean (M ± SE) of three replications. Means within the same column of each protein followed by different letters are significantly different at P<0.05.

### Detection of three ATP hydrolyzing enzymes in *H. zea*

**Figure 1:**
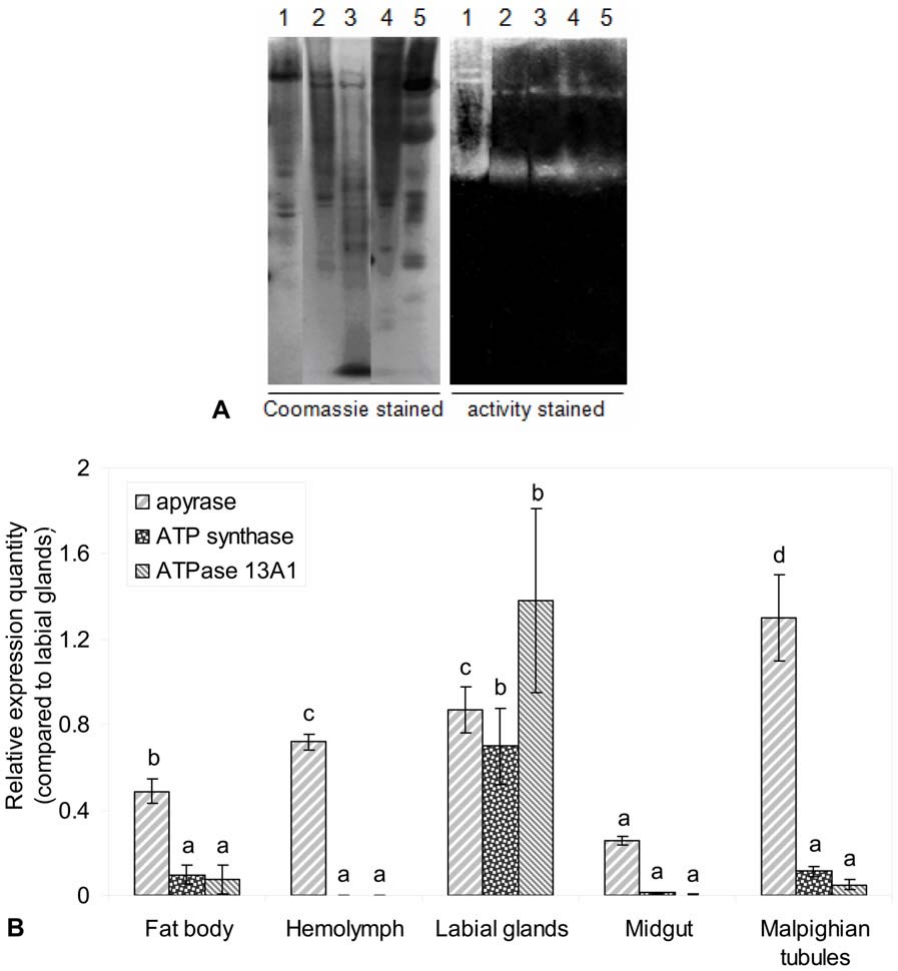
Activities and expressions of ATP hydrolyzing enzymes in *H. zea* tissues. **A**, in-gel ATP hydrolysis activity test among different tissues of *H. zea*. Lane 1–5, total crude homogenates from labial glands, fat body, hemolymph, Malpighian tubules and midgut, respectively. **B**, the relative expression levels of three target genes among different tissues of *H. zea*. Actin was used as the internal reference. Values are expressed as mean ± SE (n = 3). Significance was tested for each gene separately. Different letters above each bar indicate statistical difference determined by ANOVA analysis followed by the Duncan's Multiple Range Test (P<0.05).

The in-gel ATP hydrolysis assay showed that ATP hydrolyzing enzymes are ubiquitous among tissues. Although the same amount of crude homogenate (50 µg) was loaded on the gels, the activity bands were much stronger in labial glands than from other tissues (Figure 1A). Then, a publicly available *Heliothis virescens* EST database (http://www.uky.edu/Ag/Entomology/entdept/faculty/bwebb/heliothisvirescensests.tfa) and NCBI BLAST databases were used to identify potential ATP hydrolyzing enzymes in *H. zea*. It was noted that the ATP synthase and ATPase 13A1 proteins were highly conserved among insect species. Partial cDNAs of ATP synthase and ATPase 13A1 were synthesized using specific primers designed directly according to the nucleotide sequences (Contig number: HZ0370561 for ATP synthase and HZ0402662 for ATPase 13A1) of the closely related species, *H. virescens*, that encoding amino acids in conserved regions, which were 5′-CCATCCTAAATGCCCTTGAA-3′ (sense) and 5′-TCACCGATGACGTTGATGAT-3′ (anti-sense) for ATP synthase, 5′-TTTCCCACCTGATTACCGAATT-3′ (sense) and 5′-CCCTCGCCGAACAGAATG-3′ (anti-sense) for ATPase 13A1. However, only degenerate primers (sense: 5′-GCTATGACCCTGggnaaycayga-3′, anti-sense: 5′-CACCGTGATGCTGccnavrtaytt-3′), which were designed by alignment of apyrase amino acid sequences derived from other insects worked well for synthesizing the *H. zea* apyrase.

### Expression of three ATP hydrolysis-related genes in different tissues

Quantitative real-time PCR (qRT-PCR) was used to assess gene expression levels in labial glands and to determine the best RNA sample sources of apyrase, ATP synthase and ATPase 13A1 in *H. zea*. The relative expression of ATP synthase and ATPase 13A1 genes was highest in labial glands, where they were 6.2- and 18.0-fold higher respectively than they were in Malpighian tubules and fat body. There was no difference in the expressions of these two genes among the other tissues (P>0.05). The relative apyrase level was highest in Malpighian tubules, which was 1.5-fold higher than it was in the labial glands, and there was no difference between the levels in labial glands and hemolymph (P>0.05) (Figure 1B).

### Sequence analysis

To investigate the function of these enzymes in mediating defense, the complete cDNAs of labial gland apyrase, ATP synthase and ATPase 13A1 were cloned and expressed. Sequencing showed that their complete cDNAs consisted of 2,102, 1,789 and 4,171 bp respectively, with open reading frames of 1,641, 1,551 and 3,483 nucleotides, respectively. They encode proteins of 546, 516 and 1160 amino acids residues, and their calculated theoretical molecular masses are 61.2, 55.2 and 130.2 kDa, respectively (Figure S1, S2, S3). The sequences were deposited in GenBank (accession numbers: HM569605, HM156739 and HQ184468). Besides, the presence of N-terminal signal peptide was predicted in three amino acid sequences, since it was proved to be functional in translocating proteins across membranes to extracellular space [31], which indicates the potential secretion of the ATP hydrolyzing enzymes from the labial glands to the saliva. Although there was no signal peptide in the ATP synthase and ATPase 13A1 proteins, one was present in apyrase (Figure S1). Additionally, ATPase 13A1 and ATP synthase were diagnosed as non-classically secreted proteins by using Secretome 2.0 (http://www.cbs.dtu.dk/services/SecretomeP/), with NN-scores of 0.767 and 0.492 respectively. An NN-score above 0.5 indicates a high probability of the protein being secreted.

**Figure 2:**
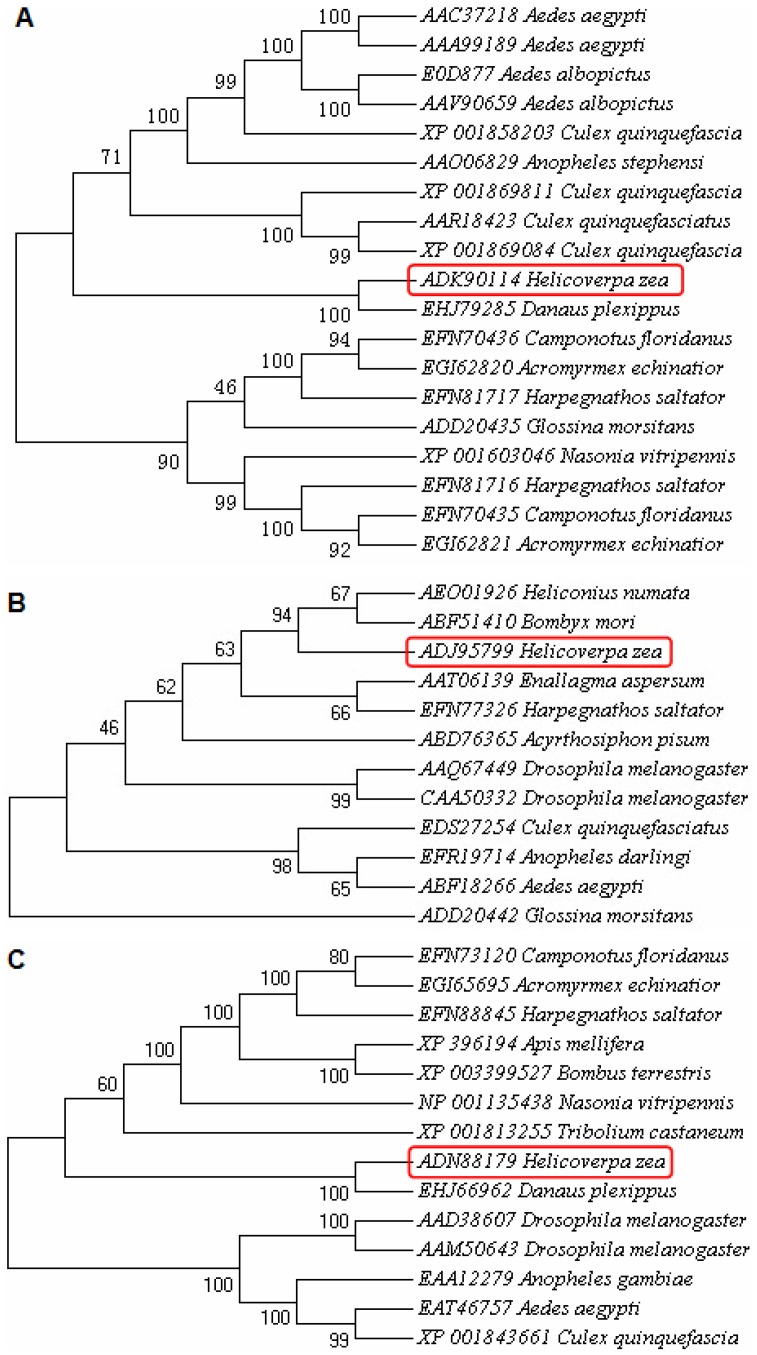
The molecular evolutionary trees of target proteins with amino acid sequences from other insect species. **A**, **B**, **C** represent the evolutionary trees for *H. zea* apyrase, ATP synthase and ATPase 13A1, respectively. The corresponding GeneBank accession numbers for *H. zea* apyrase, ATP synthase and ATPase 13A1 are ADK90114, ADJ95799 and ADN88179, respectively.

BLASTs showed that the deduced amino acid sequence of *H. zea* apyrase shares 53% identity with *Danaus plexippus* apyrase (EHJ79285) and 40% identity with *Culex quinquefasciatus* apyrase (EDS28861); *H. zea* ATP synthase shares 96% identity with *Bombyx mori* ATP synthase (ABF51410); and *H. zea* ATPase 13A1 shares 86% identity with *Danaus plexippus* ATPase 13A1 (EHJ66962). Phylogenetic trees (Figure 2) show their relationships with the enzymes from other insect species.

### Expression of ATP hydrolyzing enzymes in a heterologous system

**Figure 3:**
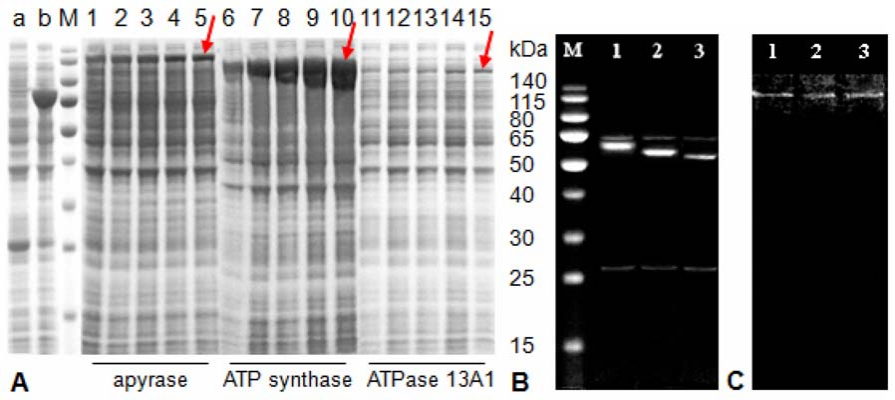
SDS-PAGE analyses of protein expression and purification. **A**, expression time-courses of the fusion proteins in *E. coli*. Lane a, control 1, total cellular proteins from *E. coli* Rosetta 2 (DE3) after being induced by 1.0 mM isopropyl β-D-1-thiogalactopyranoside (IPTG) for 2.5 h. Lane b, control 2, expression of empty pET-43.1b(+) vector protein after being induced by 1.0 mM IPTG for 2.5 h. Lane 1–5, 6–10 and 11–15 show the expressions of fusion apyrase, ATP synthase and ATPase 13A1 after being induced by 1.0 mM IPTG for 2, 3, 4, 5 and 6 h, respectively. **B**, SDS-PAGE analysis of purified apyrase (lane 1), ATP synthase (lane 2) and ATPase 13A1 (lane 3) from *E. coli*. Lane M, protein molecular weight markers. **C**, native PAGE analysis of purified apyrase (lane 1), ATP synthase (lane 2) and ATPase 13A1 (lane 3). Arrows represent the locations of target proteins.

Because the insert size that a vector could carry was limited and the larger sizes of partial ATPase 13A1 were not tolerated, and also in order to obtain good cDNAs synthesis efficiency, partial cDNAs encoding the ATP hydrolyzing enzymes were expressed in a bacterial system. The molecular masses of expressed fusion proteins showed agreement with the sizes predicted from sequence data, which were 118.4, 113.0 and 110.2 kDa for apyrase, ATP synthase and ATPase 13A1, respectively. Corresponding bands were not found in total cellular proteins from host bacteria and induced culture containing empty pET-43.1b(+) vector (Figure 3A). SDS-PAGE of purified proteins indicated that the molecular masses were 60.0, 54.5 and 51.7 kDa for apyrase, ATP synthase and ATPase 13A1, respectively (Figure 3B). Figure 3B also shows two bands (62.8 and 26.3 kDa) after the secondary purification that may be impurities.

ATP hydrolysis activity was tested after each purification step to estimate the enzymatic yields (Table 1). The specific activities varied significantly after each purification step (P<0.05). After the last purification step, the specific activities increased 1.4-, 1.9- and 1.8-fold with 111.9, 231.5 and 70.4% yield for apyrase, ATP synthase and ATPase 13A1, respectively (Table 1). Both protein concentration and total activity differed significantly between the first and the third purification steps for all proteins (P<0.05). The total activities of apyrase, ATP synthase and ATPase 13A1 were 1.5-, 2.3- and 2.4-fold higher in the final step, respectively. Additionally, the impurity proteins showed no activity.

### Plant responses to purified ATP hydrolyzing enzymes

Plants rely on different defense signaling pathways to evade attack. One is mediated by the salicylic acid (SA) pathway, which plays a role in the resistance to pathogens by inducing pathogenesis-related proteins (PR) and other genes like phenylalanine ammonia lyase (PAL) [32], [33]. The second is the jasmonic acid pathway (JA) [34]. When plants encounter herbivory, JA is released, and defense genes, such as proteinase inhibitor 2 (PIN2), arginase (ARG), polyphenol oxidase (PPO) and threonine deaminase (TD) are activated [35]–[37]. Third is the ethylene pathway which plays a role in regulating defenses through a complex network that includes JA, SA and abscisic acid that enables the plant to fine-tune its responses [38]. Osmotin (OSM), an antifungal cytotoxic agent, is transcriptionally activated by abscisic acid and ethylene. It functions as another pathogenesis-related protein [39].

In this study, purified apyrase, ATP synthase and ATPase 13A1 with similar ATP hydrolysis activity of 74.3 pmol·L^−1^·min^−1^, which was equal to the total activity of saliva collected from ten *H. zea* (Table 1), were each applied separately to a 0.3 cm^2^ wound site on the tomato leaf. In addition, dose effects of each enzyme were studied to see if the proteins themselves would cause different influences on plants, since our preliminary tests with boiled inactive commercial apyrase (New England Biolabs, Inc.) showed that it had a strong necrotic effect and on defense genes (Figure S4), and the total ATP hydrolysis activity of active commercial apyrase was 23.9-fold higher than the total ATP hydrolysis activity of saliva collected from ten *H. zea*.

**Figure 4:**
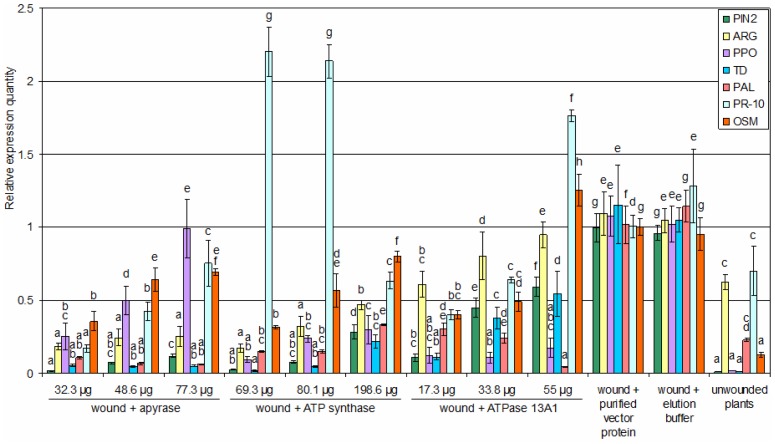
The relative expression levels of defense genes among tomato leaves after different treatments. Total RNAs were extracted from tomato leaves after 24 h of different treatments. Different quantities of proteins with the same total ATP hydrolysis activity of 74.3 pmol·L^−1^·min^−1^ were indicated. Ubiquitin was used as the internal reference. The expression level of each gene was normalized to the level in treatment with purified pET-43.1b(+) vector protein, which contain the impure protein only. Values are expressed as mean ± SE (n = 3). Significance was tested for each gene separately. Different letters above each bar indicate statistical difference determined by ANOVA analysis followed by the Duncan's Multiple Range Test (P<0.05).

The relative expression of PIN2, ARG, PPO, TD and OSM genes decreased after 24 h treatment with ATP hydrolyzing enzyme (Figure 4). PAL expression was suppressed by lower specific activities of apyrase and ATPase 13A1, but was higher with lower specific activities of ATP synthase. The results for the PR-10 genes were reversed (Figure 4). Compared to the purified pET-43.1b(+) vector protein treatment control, expression levels of PIN2, ARG, PPO, TD and PAL were inhibited significantly after most enzyme treatments (P<0.05), except for the effects of the lowest specific activities of ATPase 13A1 and apyrase on ARG and PPO genes, respectively (P>0.05). At the same time, PR-10 and OSM genes were expressed at higher levels after being treated with the lowest specific activity of ATPase 13A1 (P<0.05). Compared to the control treatments, PR-10 expression was induced after being treated with higher activities of ATP synthase (P<0.05). There was no difference between the relative expression levels of all the tested genes after being treated with the impure protein (purified vector protein) and elution buffer (P>0.05), but they were both higher than the unwounded control (P<0.05).

### Glandular trichome production

**Figure 5:**
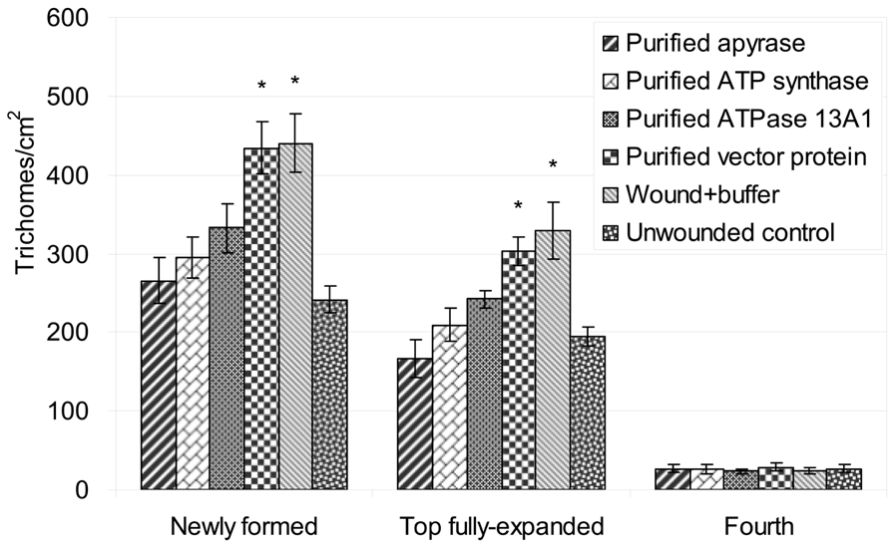
Trichome productions in tomato leaves after 10 days of different treatments. Values are expressed as mean ± SE (n = 5). Asterisks indicate statistical difference determined by ANOVA analysis followed by the Duncan's Multiple Range Test (P<0.05).

To determine the effects of the enzymes on Type VI glandular trichomes in leaves, trichomes in newly formed, top fully-expanded, and the treated fourth leaves were counted 10 days after treatment with the highest protein concentration. The wounding significantly elicited glandular trichome production in newly formed and top fully-expanded leaves (P<0.05). Although purified apyrase, ATP synthase and ATPase 13A1 had different effects on trichome production in new leaves (Figure 5), there was no significant difference between the trichome density in new leaves of unwounded plants, and of plants after treatments with purified proteins (P>0.05). There was no difference between impure protein (purified vector protein) and buffer treatments after wounding (P>0.05). The trichome densities in the fourth leaves that had been treated were similar regardless of treatments.

## Discussion

Herbivore oral secretions are important recognition cues perceived by plants [2], [3], [7], [9], [11], [40]. Their impact on defenses is determined by the relative balance between components that elicit vs. those that suppress defenses. Our initial studies on saliva together with 2D SDS-PAGE and in-gel ATP hydrolysis activity indicated that there are several proteins that were involved in ATP hydrolysis or ATP-binding, such as myosin, actin, type I secretion membrane fusion protein, kinesin, sulfate adenyltransferase, heat shock protein, ATP-binding cassette transporters, etc. However, so far, only apyrase has been verified to be present in saliva by western blot analysis (Figure S5). Based on the results of relative expression levels of apyrase, ATP synthase and ATPase 13A1 in different tissues, labial glands are the tissue these three *H. zea* ATP hydrolyzing enzymes mainly work and labial glands were used as the source of RNA samples to isolate and sequence. The high expression level of apyrase in Malpighian tubules suggests that it might play a role in the excretory and osmoregulatory systems in *H. zea*.

In the past it was believed that an N-terminal signal peptide was required for extracellular secretion of proteins [31]. Although the sequence analysis shows that there is no signal peptide in the sequence of ATPase 13A1, it possesses a significant NN-score of 0.767. However, ATP synthase had a borderline NN-score of 0.492 for non-classically secreted proteins. So, the *H. zea* ATPase 13A1 and ATP synthase may be exported through a non-classical secretory pathway, which could be any one of the four methods reported for leaderless secretion [41]–[44]. Identification analyses of the secretion of these two proteins should be studied in the future.

In this study, plant responses were determined after applying the purified expressed apyrase, ATP synthase and ATPase 13A1 to tomato leaves after 24 h treatment. The relative expressions of PIN2, ARG, PPO, TD, PAL, PR-10 and OSM genes, which are associated with different defense pathways, were analyzed. The results demonstrated that all the seven genes were induced significantly by wounding (P<0.05). However, treatments with different protein concentrations of purified apyrase, ATP synthase and ATPase 13A1 at the same total ATP hydrolysis activity of 74.3 pmol·L^−1^·min^−1^ after wounding suppressed the expressions of PIN2, ARG, PPO and TD genes significantly (P<0.05), which indicated that the ATP hydrolyzing enzymes inhibited the JA pathway (P<0.05), especially at higher specific activities. As far as the protein concentrations of purified apyrase, ATP synthase and ATPase 13A1 are concerned, the higher concentrations with lower specific activities weakened their effects on the JA pathway in wounded leaves, and accordingly, restored this defense response. Interestingly, the PAL and PR-10 genes, which both are associated with the SA pathway, had reversed reactions to these enzymes, that is, expression of PAL was suppressed by lower specific activities of apyrase and ATPase 13A1, but by higher specific activities of ATP synthase. As for PR-10 gene levels, they were totally different. The difference between the gene responses to ATP synthase and to other two proteins might be caused by the dual function of both hydrolysis and synthase activity in ATP synthase. Because of the limitations in our assay for ATP hydrolysis activity, it could not be determined if there was any ATP synthesized during measurement. The relative expression of PR-10 genes revealed that higher specific activities of ATP synthase and the lowest specific activities of ATPase 13A1 induced the SA pathway (P<0.05). Besides, the suppressed OSM gene expression levels by higher specific activities of these proteins suggest that these three ATP hydrolyzing enzymes possibly mediate defenses through inhibiting the ethylene pathway.

The suppression of JA-regulated defense gene expression by ATP hydrolyzing enzymes may have multiple causes. One cause is the consumption of energy (ATP) by certain specific activities of ATP hydrolyzing enzymes, because plants would not be able to complete normal physiological responses when lacking in eATP, and the results showed that not all the specific activities of these enzymes could suppress tomato plant defenses. A second cause is the upregulation of SA-dependent plant defenses (e.g., PR-10) by the ATP hydrolyzing enzymes from herbivores. There is ample evidence that such upregulation can cause suppression of JA-dependent defenses such as PDF1.2, VSP2, LOX2, AOS, AOC2, and so on [45], [46].

In this study, very different plant responses to commercial apyrase and *H. zea* apyrase were observed, which provides a cautionary note about using commercial enzymes as models. The commercial enzyme was purified from *Kluyveromyces lactis* containing a clone of the potato apyrase gene. The effects of the commercial enzyme in eliciting defense gene expression were not due to its enzymatic activity because boiling did not affect its ability to elicit defenses. Instead most likely a contaminant and/or the structural properties of the protein are responsible for the activity. Results from deduced amino acid sequence alignment of apyrase between potato (accession number: HM136581) and *H. zea* showed that they only shared 11.8% identity.

The function of glandular trichomes in defense against herbivores has been known for decades [47]. The glandular trichome production was studied after 10 days treatment with the highest concentration of each purified protein. Wounding induced the production of higher trichome densities compared to unwounded plants (P<0.05). However, after treatment with purified ATP hydrolyzing enzymes, there was no significant difference between the trichomes in wounded and unwounded plants (P>0.05). These results are consistent with the results of plant defensive genes, since the JA pathway regulates glandular trichome development in tomato [36].

Our findings show that, in addition to mediating pathogen resistance, eATP also plays a regulatory role in mediating defenses to herbivores. Plant apyrases limit the accumulation of nucleotides released during wounding and pathogen attack, and thus help terminate eATP signaling [48]. Based on our results, larvae secrete ATP hydrolyzing enzymes that suppress plant defenses and function as effectors. The overall impact of saliva on plant defenses depends upon the relative activity/balance of effectors and proteins that may possess opposing, eliciting activity. The host plant is also an important component because one salivary component, e.g., glucose oxidase, can have very different effects on induced defenses depending upon the host plant species [7], [10].

Perhaps the most intriguing finding is that herbivorous insects may employ a similar mechanism to limit host eATP accumulation and signaling as blood-feeding arthropods that use apyrase to degrade eATP. Apyrases are ubiquitous salivary proteins in blood-feeding arthropods. Mosquito salivary apyrases, as is the *H. zea* apyrase, are members of the 5′-nucleotidase family of apyrases [49]. Hence the comparatively high degree of sequence similarity of the *H. zea* salivary apyrase with mosquito apyrases suggests a broader evolutionary role for salivary apyrases than previously envisioned.

## Materials and Methods

### Insect and plant material

*H. zea* eggs were obtained from the North Carolina State University Insectary. Larvae were reared on a wheat germ and casein-based artificial diet [50]. Insects were kept at 27°C, with a 16 h photoperiod. Tomato seeds (*Solanum lycopersicum* cv. Betterboy) were grown in Pro-mix potting soil (Premier Horticulture, Quakertown, PA, USA) and fertilizer (Scotts, Marysville, OH, USA) in greenhouse with a 16 h photoperiod and were used at the four-leaf stage.

### ATP Hydrolysis Activity Assay

In-gel protein ATP hydrolysis activity tests were performed according to the method of Wittig and Schägger [51] by incubating the 8% native polyacrylamide gel, which carried the target proteins, in 270 mM glycine, 35 mM Tris, 8 mM ATP, 14 mM MgSO_4_, 0.2% Pb(NO_3_)_2_, pH 8.4 for 5–10 min. The reaction was stopped by 30 min incubation in 50% methanol, 50% water, followed by 30 min incubation in water. ATP hydrolysis activities of *H. zea* saliva, expressed fusion proteins and purified protein solutions were measured using ENLITEN® ATP Assay System Bioluminescence Detection Kit (Promega Corp.) on a FLUOstar OPTIMA fluorometer (BMG LABTECH, Offenburg, Germany). To obtain eATP from tomato leaves, 200 mg leaves from each plant were infiltrated under vacuum for 15 min in ATP-free water according to the method of Terry and Bonner [52] with slight modifications. The leaves were dried on filter paper and the rolled leaves were centrifuged in a 30 K MWCO Nanosep® Centrifugal Devices (Pall Corp.) for 20 min at 1,000 g. About 100 µl extracellular fluids from each plant containing eATP were collected and were used for testing *H. zea* saliva ATP hydrolysis activity.

### RNA Extraction and cDNA Synthesis

To isolate the *H. zea* labial glands, Malpighian tubules, midgut and fat body, larvae were placed on ice and chilled until flaccid, and then, the larvae were fastened ventral side up on a dissecting pan with two steel pins punctured through both ends. *H. zea* tissues were collected and washed with distilled water separately after cutting open its epidermis using dissecting scissor. *H. zea* hemolymph samples were collected from separate individual larvae. All the *H. zea* tissues were frozen with liquid nitrogen and kept in −80°C freezer before use. Total RNA was extracted from *H. zea* tissues and tomato leaves using RNeasy Plus Mini Kit and QIAshredder (Qiagen, Valencia, CA, USA). First-strand cDNAs were synthesized using SMARTer™ RACE cDNA Amplification Kit (Clontech, Mountain View, CA, USA) and High-Capacity cDNA Reverse Transcription kit (Applied Biosystems, Foster City, CA, USA), respectively. Degenerate primers for apyrase were designed using online software http://bioinformatics.weizmann.ac.il/blocks/codehop.html. To obtain full-length cDNAs, Gene-specific primers (GSPs) for rapid amplification of cDNA ends (RACE) were designed using primer premier 5.00 for Windows (http://www.PremierBiosoft.com). The PCR products were separated by 1.0% agarose gel electrophoresis and were stained with SYBR® safe DNA gel stain (Invitrogen, USA).

### Cloning, Sequencing and Sequence Analysis

The PCR products were purified using the E.Z.N.A.™ Gel Extraction Kit (Omega Bio-Tek, Inc.), and then cloned into pGEM®-T easy vector (Promega Corp.). The ligation reactions were used for transformations with *E. coli* JM109 competent cells. Recombinant plasmids were extracted using FastPlasmid™ Mini Kit (5Prime, Inc.) and were sent to Nucleic Acid Facility (406 Chandlee Lab, Penn State University, PA, USA) for sequencing. Blast research was performed at http://www.ncbi.nlm.nih.gov/blast/. ExPASy Proteomics Server (http://cn.expasy.org/tools/pi_tool.html) was used to compute molecular mass of deduced protein sequences. The signal peptide was predicted by SignalP 4.0 server program (http://www.cbs.dtu.dk/services/SignalP) [53].

### Prokaryotic Expression

Partial sequences of the *H. zea* labial gland apyrase, ATP synthase and ATPase 13A1, which contain the conserved active domains for ATP hydrolysis activities (Figure S1, S2, S3), were subcloned into the pET-43.1b(+) vector (Novagen, USA) and transformed into *E. coli* Rosetta 2 (DE3) competent cells (Novagen, USA). The transformants harboring the recombinant plasmid were confirmed by DNA sequencing and restriction endonuclease analysis. To express the recombinant protein, a single colony was inoculated into 2 ml LB medium containing 100 µg·ml^−1^ ampicillin and was incubated with shaking at 37°C until the OD_600_ reaches 1.0. The cells were collected by centrifugation and were resuspended in 50 ml fresh medium plus 200 µg·ml^−1^ ampicillin. Then, the culture was incubated in a 250 ml Erlenmeyer flask with shaking at 37°C until OD_600_ reaches 0.6. The fusion protein was induced with 1.0 mM IPTG and 2% glucose and continued shaking at 30°C for 2–6 h. Cells were harvested by centrifugation (4°C, 5,000 g, 5 min). The results of gene expression time-courses were analyzed using 12% NuPAGE® Novex® Bis-Tris Mini Gels and XCell SureLock™ Mini-Cell rig (Invitrogen, USA). The gel was stained with SimplyBlue™ SafeStain (Invitrogen, USA). The protein molecular masses were calculated by the Gel Doc™ XR System and Quantity One 1-D Analysis Software (Bio-Rad Laboratories, Hercules, CA, USA) after scanning.

### Protein Purification

According to the protocol supplied by Novagen, and in order to obtain the optimal expressed protein quantities and to maintain protein activities, protein purification was performed after 2.5 h induction. The expressed fusion proteins were purified from *E. coli* using S·Tag™ Thrombin Purification Kit (Novagen, USA) with 3 K and 10 K MWCO Nanosep® Centrifugal Devices (Pall Corp.) for concentration and changing the buffer. All the proteins were purified twice. ATP hydrolysis activity was tested during the secondary purification.

### QRT-PCR

The relative transcription levels of mRNA in tomato and insect samples were examined with housekeeping genes ubiquitin and actin as references, respectively, and Power SYBR Green PCR Master Mix utilizing the ΔΔ*CT* method [54] on a 7500 Fast Real-Time PCR System (Applied Biosystems, Foster City, CA, USA). Thermal cycling conditions were: 10 min at 95°C, followed by 40 cycles of 15 s at 95°C, 60 s at 60°C. The efficiency of PCR amplification for GSPs was analyzed by one cDNA sample with five serial dilutions, three technical replications. Standard curve for each gene was made depending on the Ct values at different cDNA concentrations. GSPs with approximate 100% efficiency (R^2^>0.97) were chosen for qRT-PCR test (Table S1). Melting curve analysis and gel electrophoresis both showed the unique target gene for each sample. Data were statistically analyzed using analysis of variance (ANOVA), and the means were separated by Duncan's Multiple Range Test for significance (P = 0.05) by using SPSS 12.0 for Windows (SPSS, Inc., Chicago, IL, USA).

## Supporting Information

Figure S1 **Nucleotide and deduced amino acid sequences of labial gland apyrase from** ***H. zea*** **.**
(DOC)

Figure S2 **Nucleotide and deduced amino acid sequences of labial gland ATP synthase from** ***H. zea*** **.**
(DOC)

Figure S3 **Nucleotide and deduced amino acid sequences of labial gland ATPase 13A1 from** ***H. zea*** **.**
(DOC)

Figure S4 **The relative expression levels of defense genes among tomato leaves after 24 h of different treatments.**
(DOC)

Figure S5 **Western blot analysis of** ***H. zea*** **apyrase with peptide antibody (DGGDGFSMFRDGKQ).**
(DOC)

Table S1 Primers used for real-time PCR assays of relative gene expression.
(DOC)
