# Supplementary material for: ATP Hydrolyzing Salivary Enzymes of Caterpillars Suppress Plant Defenses
Source: PLoS One. 2012 Jul 25;7(7):e41947. doi: 10.1371/journal.pone.0041947 (PMC3405022; doi:10.1371/journal.pone.0041947)
Supplement: Figure S1 — Nucleotide and deduced amino acid sequences of labial gland apyrase from H. zea . (DOC) [file pone.0041947.s001.doc]

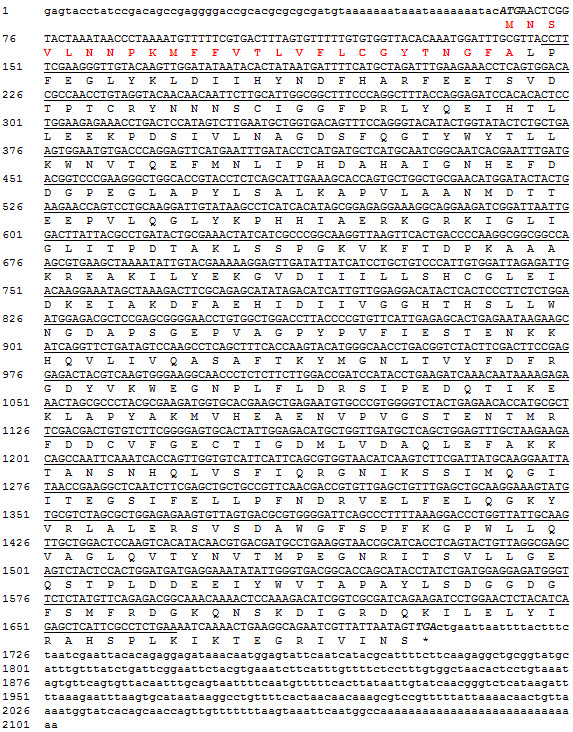


**Figure S1. Nucleotide and deduced amino acid sequences of labial gland apyrase from *H*. *zea*.** The 5′ and 3′ untranslated regions (UTRs) were lowercased. The start and stop codons were highlighted in bold italic. The amino acid sequence of signal peptide was shown in red. The expressed region was underlined.
