# Supplementary material for: ATP Hydrolyzing Salivary Enzymes of Caterpillars Suppress Plant Defenses
Source: PLoS One. 2012 Jul 25;7(7):e41947. doi: 10.1371/journal.pone.0041947 (PMC3405022; doi:10.1371/journal.pone.0041947)
Supplement: Figure S3 — Nucleotide and deduced amino acid sequences of labial gland ATPase 13A1 from H. zea . (DOC) [file pone.0041947.s003.doc]

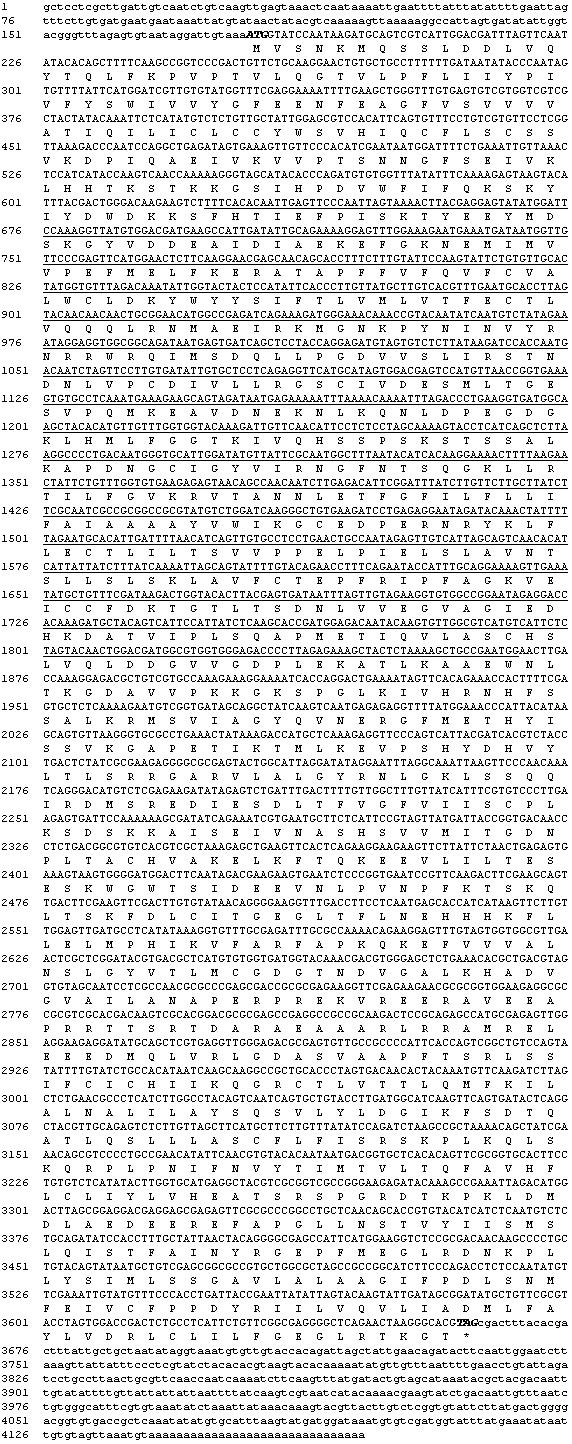


**Figure S3. Nucleotide and deduced amino acid sequences of labial gland ATPase 13A1 from *H*. *zea*.** The 5′ and 3′ UTRs were lowercased. The start and stop codons were highlighted in bold italic. The expressed region was underlined.
