# Supplementary material for: ATP Hydrolyzing Salivary Enzymes of Caterpillars Suppress Plant Defenses
Source: PLoS One. 2012 Jul 25;7(7):e41947. doi: 10.1371/journal.pone.0041947 (PMC3405022; doi:10.1371/journal.pone.0041947)
Supplement: Figure S4 — The relative expression levels of defense genes among tomato leaves after 24 h of different treatments. (DOC) [file pone.0041947.s004.doc]

**Figure S4. The relative expression levels of defense genes among tomato leaves after 24 h of different treatments.** Ubiquitin was used as the internal reference. The expression level of each gene was normalized to the level in unwounded control plants. Values are expressed as mean ± SE (n = 3). Significance was tested for each gene separately. Different letters above each bar indicate statistical difference determined by ANOVA analysis followed by the Duncan′s Multiple Range Test (P < 0.05).
