# Supplementary material for: ATP Hydrolyzing Salivary Enzymes of Caterpillars Suppress Plant Defenses
Source: PLoS One. 2012 Jul 25;7(7):e41947. doi: 10.1371/journal.pone.0041947 (PMC3405022; doi:10.1371/journal.pone.0041947)
Supplement: Figure S5 — Western blot analysis of H. zea apyrase with peptide antibody (DGGDGFSMFRDGKQ). (DOC) [file pone.0041947.s005.doc]

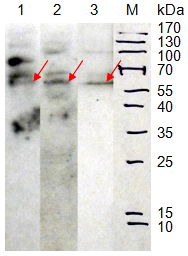


**Figure S5. Western blot analysis of *H*. *zea* apyrase with peptide antibody (DGGDGFSMFRDGKQ).** Lane 1-3, total proteins from *H*. *zea* saliva, labial glands and purified expressed apyrase from *E*. *coli*, respectively. Arrows represent the locations of target proteins.
