# Supplementary material for: ATP Hydrolyzing Salivary Enzymes of Caterpillars Suppress Plant Defenses
Source: PLoS One. 2012 Jul 25;7(7):e41947. doi: 10.1371/journal.pone.0041947 (PMC3405022; doi:10.1371/journal.pone.0041947)
Supplement: Table S1 — Primers used for real-time PCR assays of relative gene expression. (DOC) [file pone.0041947.s006.doc]

| Gene | Description | Accession No. | Forward/reverse sequence (5′ to 3′) | note |
| --- | --- | --- | --- | --- |
| PIN2 | Wound inducible proteinase inhibitor 2 | K03291 | GGATTTAGCGGACTTCCTTCTG/ATGCCAAGGCTTGTACTAGAGAATG | tomato |
| ARG | Arginase | AY656838 | TGGTGAAGGTGTAAAGGGCGTGTA/TTACCAGCTTCGCAGCAACCATTG | tomato |
| PPO | Polyphenol oxidase E | Z12837 | AACCCTTACCGTGTGAAAGTCCGT/TAACTCCCTGCAAACTCCGCCTTA | tomato |
| TD | Threonine deaminase | M61915 | CTGGAGCTGCAGCCTATTGTGAAT/TGCCTGAACCTAACCCTGCTAACT | tomato |
| PAL | Phenylalanine ammonia lyase | M83314 | TTCGAGTTGCAGCCTAAGGAAGGA/ATAGCAGCAGCCTCAATCTGACCA | tomato |
| PR-10 | Pathogenesis related protein family 10 | Y15846 | TGTAGGCAGAAGAGAGAATCATGAA/CAGAAGGATTCGCGAGAAGGT | tomato |
| OSM | Osmotin | AF093743 | AGTACGCCTTGGACCAGTTTAGCA/CACATGGACCTTGGGTGCAACAAT | tomato |
| UBI | Ubiquitin | X58253 | GCCAAGATCCAGGACAAGGA/GCTGCTTTCCGGCGAAA | tomato |
| APY | Apyrase | HM569605 | ATATTATCATCCTGCTGTCCCATT/TGATGCTTCTTATTCTCAGTGCTC | insect |
| SYN | ATP synthase | HM156739 | TGCTGTAGGTTACCAACCCACCTT/AGTCAAGTCATCAGCAGGCACGTA | insect |
| ASE | ATPase type 13A1 | HQ184468 | TTTCCCACCTGATTACCGAATT/CCCTCGCCGAACAGAATG | insect |
| ACT | Actin | AF286061 | CGTTGCCCTGAGGCTCTCT/GATGCCGTTGGCTTCCAT | insect |

**Table S1.** Primers used for real-time PCR assays of relative gene expression
